# Bulky Cation-Modified Interfaces for Thermally Stable Lead Halide Perovskite Solar Cells

**DOI:** 10.1021/acs.chemmater.4c03468

**Published:** 2025-05-15

**Authors:** Sakshi Sharma, Carlo A. R. Perini, Courtney Brea, Sarah Wieghold, Ruipeng Li, Letian Dou, Antonio Facchetti, Guoxiang Hu, Juan-Pablo Correa-Baena

**Affiliations:** † School of Materials Science and Engineering, 1372 Georgia Institute of Technology, Atlanta, Georgia 30332, United States; ‡ Advanced Photon Source, 1291 Argonne National Laboratory, Lemont, Illinois 60439, United States; § National Synchrotron Light Source II, 8099 Brookhaven National Laboratory, Upton, New York 11973, United States; ∥ Davidson School of Chemical Engineering, 311308 Purdue University, West Lafayette, Indiana 47907, United States; ⊥ Birck Nanotechnology Center, Purdue University, West Lafayette, Indiana 47907, United States; # School of Chemistry and Biochemistry, Georgia Institute of Technology, Atlanta, Georgia 30332, United States; ∇ Department of Chemistry, Northwestern University, Evanston, Illinois 60208, United States

## Abstract

Charged conjugated organic molecules offer promising prospects for reducing nonradiative recombination at interfaces in perovskite solar cells, while protecting the active layer from moisture. However, several studies have shown that the heat-induced diffusion of these cations leads to irreversible solar cell degradation. Passivation molecules for perovskite can reconstruct the film surface into lower-dimensional phases when exposed to thermal stress, impeding charge extraction and affecting the photoconversion efficiency (PCE) of devices. In this work, we study how molecular interactions between passivation molecules and 3D CsFAPbI_3_ perovskite impact stability and charge extraction at the perovskite/hole transport layer interfaces. Two model π-conjugated molecules are studied: 2-([2,2′-bithiophen]-5-yl)­ethan-1-aminium iodide (2TI) and 2-(3‴,4′-dimethyl-[2,2′:5′,2″:5″,2‴-quaterthiophen]-5-yl)­ethan-1-ammonium iodide (4TmI). We demonstrate that the speed of surface layer reconstruction under thermal stress can be controlled by the cation size and correlate these structural changes with the solar cell performance and stability. Devices treated with 2TI and 4TmI achieve PCEs over 21% and maintain their performance under thermal stress. Our findings demonstrate that thermal stability in PSCs can be achieved via the design engineering of passivation agents, offering a blueprint for developing next-generation passivation molecules.

## Graphical abstract

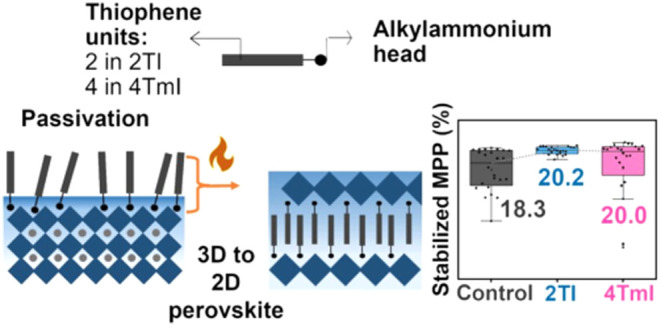

## 1 Introduction

Perovskite solar cells (PSC) have garnered significant interest in the photovoltaic research community thanks to their excellent optical and electrical properties such as long carrier diffusion length, low exciton binding energy, high absorption coefficient, and high defect tolerance in addition to easy solution processability.^1^ The perovskite structure has an ABX_3_ composition, where “*A*” is a monovalent cation such as Cs or organic CH­(NH_2_)_2_^+^ (formamidinium cation, FA^+^), “B” is a divalent metal cation such as Pb^2+^, and “X” is a halogen anion.^2^ Therefore, the perovskite structure provides compositional flexibility that in turn controls the structure and thus, the optoelectronic properties of the material.^3,4^ To date, the highest certified efficiencies for a single junction PSC and for perovskite/Si tandem solar cells are 26.7 and 34.6%, respectively.^5^ These impressive efficiencies have been achieved mainly through interface passivation between the charge transport and the perovskite layers.^6–8^ Interfaces give rise to dangling bonds or lattice imperfections, which can serve as trap states to capture photogenerated carriers and become centers for nonradiative recombination.^9^ Further, interfaces determine whether the photogenerated carriers in the perovskite can be transferred to the electron and hole transport layers. The energy band alignment between the perovskite and charge transport layers is crucial as band energy mismatch can lead to carrier accumulation and recombination at the interface, affecting the open-circuit voltages (*V*_OC_).^10,11^ So far, researchers have focused on the development of polymers, organic molecules, and low-dimensional materials to mitigate this undesirable surface recombination.^8,12–15^

Bulky organic cation molecules, among which aromatic amines offer some unique advantages, are the most widely used surface passivation agents in perovskite solar cells.^16,17^ These bulky cations are too big to fit in the 3D perovskite lattice, and their molecular structures consist of functional groups that act as an anchor on the perovskite surface and an organic backbone present as either aromatic with multiple ring moieties, or aliphatic chains.^18^ By passivating surface and grain boundary defects, nonradiative recombination can be suppressed.^19^ Molecular structures of passivation agents also influence their bandgap and conductivity, which co-determines optoelectronic performances in perovskite devices and constitutes the focus of upcoming avenues on rational molecular design strategies.^20–23^ While most of the work in the literature has so far focused on defect passivation and reduction of nonradiative recombination, some reports have started to point at the thermal instability introduced by molecules used for surface passivation.^24–26^ The stability of passivation layers is crucial to protect the perovskite from degradation during long-term exposure to environmental stressors such as heat, moisture, or oxygen, to promote their long-term viability.^6^ Under the high external temperatures in which solar cells operate, potentially detrimental interfacial changes have been reported by commonly used molecules.^24–26^ In our previous work, we investigated the effect of 200 h of thermal stress on devices treated with commonly explored phenethylammonium iodide (PEAI) passivation layers and reported drops in current and voltage in passivated devices due to heat-induced cation diffusion.^27^ We showed that bulky cations may mobilize into the 3D perovskite structure by partially replacing the “A” cation sites, leading to reconstruction to lower dimensionality phases.^28^ These lower dimensionality phases typically consist of a spacer layer of the organic cations separating the *n* layers of corner-sharing lead halide octahedra. These 2D layers have been shown to exhibit hydrophobicity and to prevent ion migration-related hysteresis effects in 3D perovskite devices.^29–31^ However, the 2D layers exhibit wider bandgaps, and their insulating character can prevent charge carrier extraction at the perovskite/charge transport layers interface.^32,33^ Together, this organic/inorganic system functions as a multiple quantum well system, where the organic spacer “barrier” hinders the extraction and transport of photogenerated carriers from the 3D perovskite film.^34,35^

Considering these factors, research is warranted to explore alternative materials that offer conjugated functionalities to maintain charge transfer,^35^ along with the ability to withstand high temperatures by forming a robust capping layer of strongly interacting molecules. Several recent works use sulfur-containing heterocycles such as thiophene to attain the synergistic effect of defect passivation and carrier transport.^36–38^ First, thiophene derivatives such as oligo/fused thiophenes exhibit greater charge transport properties than phenylenes/acenes due to their high polarizability as well as strong π–π and S–S interactions.^39^ Second, reports suggest stabilization of the corner-sharing [PbX_6_]^4–^ octahedral framework due to formation of coordination bonds between the electron lone pair of sulfur with the electron-deficient Pb^2+^, in addition to the hydrogen bonding between the NH^_3_+^ anchoring group and I^–^.^40–43^ These interactions serve to suppress undercoordinated Pb^2+/^Pb^0^ defects and iodine vacancies. Long-chain organic ammonium cations such as 2-([2,2′-bithiophene]-5-yl)­ethan-1-ammonium iodide, (denoted as 2TI) and 2-(3‴,4′-dimethyl-[2,2′:5′,2″:5″,2‴-quaterthiophen]-5-yl)­ethan-1-ammonium iodide (denoted as 4TmI) have been used to enable defect passivation through electron-rich donor atoms and conjugation-induced charge transport at perovskite interfaces.^7,44,45^ Third, energy-level characterization of passivation layers reveals that these cations minimize the energy mismatch between the perovskite and hole transport layer (HTL) through shifts in valence band maxima at the interfaces, enabling lower carrier recombination rate and extended carrier lifetimes.^45^ Interactions of thiophene rings with perovskites have been shown to suppress grain boundary defects and undesired ion migration, thus ultimately enhancing the thermal stability of the underlying perovskite film by forming a continuous passivation layer.^46^ Finally, we note that tailoring the steric size of the passivation molecules is an opportunity to control their molecular mobility and consequently any phase changes resulting from elevated temperature exposure.

In this work, we address heat-induced performance degradation in perovskite solar cells by exploring the role of molecular design in controlling surface layer reconstruction at the perovskite/hole transport layer (HTL) interface. Using two conjugated passivation molecules, 2Tl and 4Tml, we demonstrate how their steric size governs diffusion behavior under thermal stress and explore its implications for device performance. We show that 2TI transforms into a 2D Ruddlesden–Popper (RP) phase early on, within 10 min of thermal treatment, yet maintains charge extraction to the 2,2′,7,7′-tetrakis­[*N*,*N*-di­(4-methoxyphenyl)­amino]-9,9′-spirobifluorene (Spiro-OMeTAD) HTL. In contrast, the larger 4TmI molecule forms aggregates and diffuses slowly upon thermal annealing, leading to structural phase transformations only after prolonged thermal treatment while still maintaining charge extraction. Both 2TI and 4TmI layers, when incorporated as passivation interlayers in devices, enhance the solar cells’ photoconversion efficiency (PCE) and retain the PCE enhancement even after thermal annealing of the layers. Our best device exhibits a PCE of 22.3% after thermal treatment of the 2TI-capped perovskite layer.

## 2 Results and Discussion

### 2.1 Characterization of Surface Treatments and Thin Film Properties

We prepared 4 mM solutions of 2TI and 4TmI (molecules shown in Figure 1A) dissolved in isopropyl alcohol and spin-coated them onto Cs_0.05_FA_0.95_PbI_3_ thin films (hereafter labeled CsFA, Figure S1). The films are subsequently annealed at 100 °C for varying times of 0, 10, and 40 min to understand their intercalation behavior. The films are labeled as 2TI, 0; 2TI,10; 2TI, 40, 4TmI, 0; 4TmI, 10; and 4TmI, 40, where the numbers refer to the annealing times in minutes.

**Figure 1:**
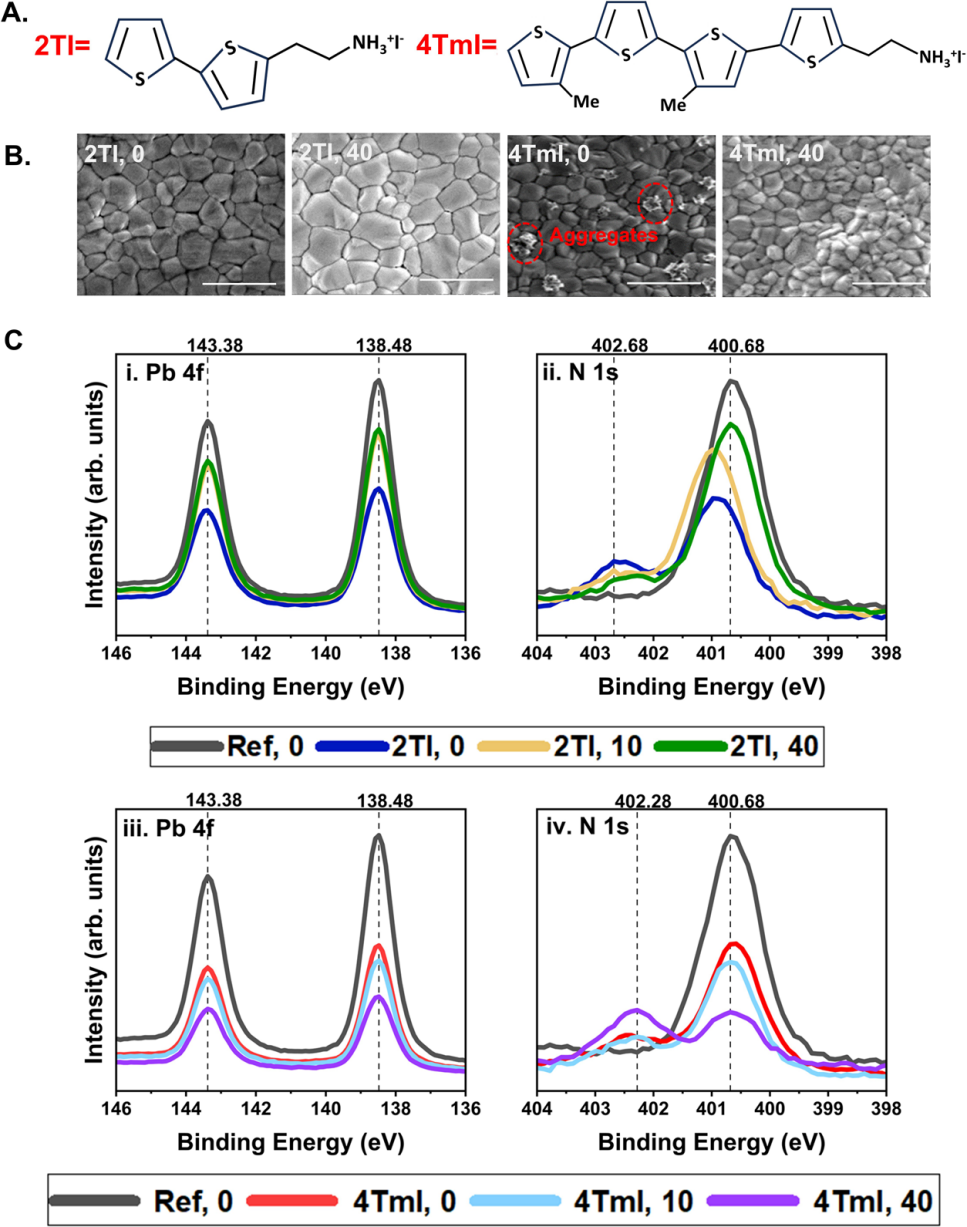
(A) Molecular structures of 2TI (left) and 4TmI (right) used in this work. (B) Film morphology seen in SEM images for unannealed and 40 min annealed films of 2TI and 4TmI. Scale bar is 1 μm. (C) XPS spectra for the reference film (black) versus passivated films- 2TI: Pb 4f (i), N 1s (ii) and 4TmI: Pb 4f (iii), N 1s (iv). In the notation 2TI,0; 2TI, 10; 2TI, 40 and 4TmI, 0; 4TmI, 10; 4TmI, 40; 0, 10, and 40 are annealing times at 100 °C in minutes.

We performed scanning electron microscopy (SEM) to observe the differences in surface morphology before and after molecule treatment and to contrast the evolution of 2TI versus 4TmI upon annealing. SEM images showing the top view of the films are presented in Figure 1B. The SEM image of the reference perovskite film is provided in Figure S2. The images indicate that surface morphology is preserved after treatment with 2TI, while treatment with 4TmI leads to the formation of clusters on the surface of the film. We attribute these clusters to the poor solubility of 4TmI forming large aggregates upon deposition. The aggregates disappear upon annealing for 40 min, suggesting heating-induced diffusion of 4TmI molecules on the surface. Surface maps of passivated perovskite films obtained using kelvin probe force microscopy (KPFM) are presented in Figure S3, comparing the contact potential difference (CPD) from as-deposited films to films annealed for 40 min at 100 °C. The CPD maps reveal that annealing redistributes 4TmI molecular clusters, homogenizing the surface potential under both dark and illuminated conditions, consistent with SEM observations of diffusion behavior of 4TmI molecules. To corroborate the formation of the capping layer at the surface, as well as to probe the compositional changes, we performed surface-sensitive X-ray photoelectron spectroscopy (XPS) measurements on the reference and surface-modified CsFA films at different annealing times. The elemental scans for N 1s and Pb 4f are presented in Figure 1C. The additional elemental scans (C 1s, S 2p, and I 3d) are reported in Figures S4 and S5. I/Pb and N/Pb elemental ratios (differentiating the N signal originating from FA versus that from the passivation cations) calculated by peak fitting the N 1s and Pb 4f scans are provided in Table S2. For both molecules, the presence of the S 2p signal (Figures S4 and S5) confirms the presence of the cations at the surface of the films. The intensities of Pb 4f signals in the XPS scans provide important insights into the diffusion behavior of the molecule under thermal stress. The Pb peak intensities at 138.48 and 143.38 eV of both as-deposited 2TI and 4TmI films (i.e., 2TI, 0 and 4TmI, 0) are suppressed compared to the reference due to the presence of the molecules deposited over the perovskite film. Upon annealing at 100 °C for 10 min, the intensity of the Pb 4f peak increases for 2TI-treated films due to greater exposure of the underlying lead halide in perovskite. In contrast, the Pb 4f intensity decreases for 4TmI-treated films, suggesting that the 4TmI molecules are progressively covering the surface, thereby hindering the detection of the lead halide signal. After 40 min of annealing, the Pb 4f signal in 2TI does not change significantly, whereas the Pb 4f signal from 4TmI continues to decline, indicating increased surface coverage by the 4TmI molecules.

In the N 1s scans, the peak at 400.68 eV, attributed to the +1/2 charged N in the N=C bond of formamidinium (FA), decreases in intensity as passivating molecules (2TI or 4TmI) are introduced atop the FA-containing perovskite layer. Contemporarily, a new peak appears at 402.68 eV, from the +1 charged N in the C–N bond in 2TI and 4TmI. In the 2TI-treated films, annealing for 10 min results in an increase in the N=C signal, which continues up to 40 min of annealing. Simultaneously, the intensity of the N–C signal from 2TI decreases with annealing. This, together with the Pb 4f intensity trends discussed above, corroborates the hypothesis of 2TI molecules penetrating the perovskite layer within 10 min of annealing. On the other hand, 4TmI-treated films do not show significant intensity changes in the first 10 min of annealing but exhibit a notable decrease in the N=C signal upon annealing for 40 min. The C–N signal originating from the 4TmI molecules follows the opposite trend, showing a notable increase in the signal only after annealing for 40 min as more 4TmI molecules diffuse on the surface.

We hypothesize that these changes in chemistry are associated with the reaction of bulky cations with the surface layer of the 3D perovskite. For 2TI, the surface compositional changes are more rapid and complete within the first 10 min of annealing. On the other hand, for the bulkier 4TmI molecules, more energy is required to separate the 4TmI aggregates and achieve uniform diffusion on the perovskite surface.

To corroborate this interpretation, we correlate the compositional changes at the surface with structural information by performing grazing incidence wide-angle X-ray scattering (GIWAXS). We performed the measurements at incidence angles (α_incident_) of 0.1 and 0.5° to probe different penetration depths for films of each variation. For an incident X-ray beam of energy 13.5 keV, α_incident_ = 0.1 and 0.5° correspond to depths of around 4 nm (referred to as “surface”) and 225 nm (referred to as “bulk”), respectively.^47^ In Figure 2, we present the sector averages of treated and untreated films, taken by radially integrating 2D GIWAXS patterns along 0 ± 20° from the vertical *q*_*z*_. The source 2D GIWAXS patterns for the different angles are presented in Figures S6 and S7. The use of sector averages allows us to enhance the signal from low-dimensional phases formed at the interface, which have scattering planes generating most of the signal parallel to the sample surface.^27^ The 1D integrated profiles presented in Figure 2 represent the surface, while diffraction from the bulk is reported in Figure S8 for different treatments and annealing times. In Figure 2, comparison of the intensity profiles of the 2TI-treated perovskite films with the pristine perovskite layer reveals a signal at *q* = 0.3 Å^–1^ that can be attributed to the low-dimensional (2T)_2_PbI_4_ Ruddlesden–Popper phase as previously identified.^34^ This signal emerges after 10 min of thermal treatment and persists, although with reduced intensity, upon prolonged annealing for 40 min at 100 °C. The lowered intensity of this signal in 2TI, 40 suggests a partial loss of crystallinity at the interface during extended annealing. For the case of 4TmI-treated interfaces, a peak is visible at *q =* 0.47 Å^–1^ at 0- and 10 min of annealing, which is assigned to 4TmI crystallites. These results are consistent with the presence of aggregates at the surface of the 4TmI, 0, 4TmI, 10 films, as observed by SEM in Figure S2. Annealing of 4TmI passivated films for 40 min leads to the emergence of two new peaks at *q =* 0.38 and 0.59 Å^–1^, which belong, respectively, to the (400) and (600) planes of the (4Tm)_2_PbI_4_ phase.^34^ These results are consistent with the XPS data, corroborating the hypothesis of 2T^+^ penetrating the perovskite layer already after 10 min of annealing, while 4Tm^+^ requiring a longer annealing time to spread on the surface and overcome the interactions between the backbones of the cations.

**Figure 2:**
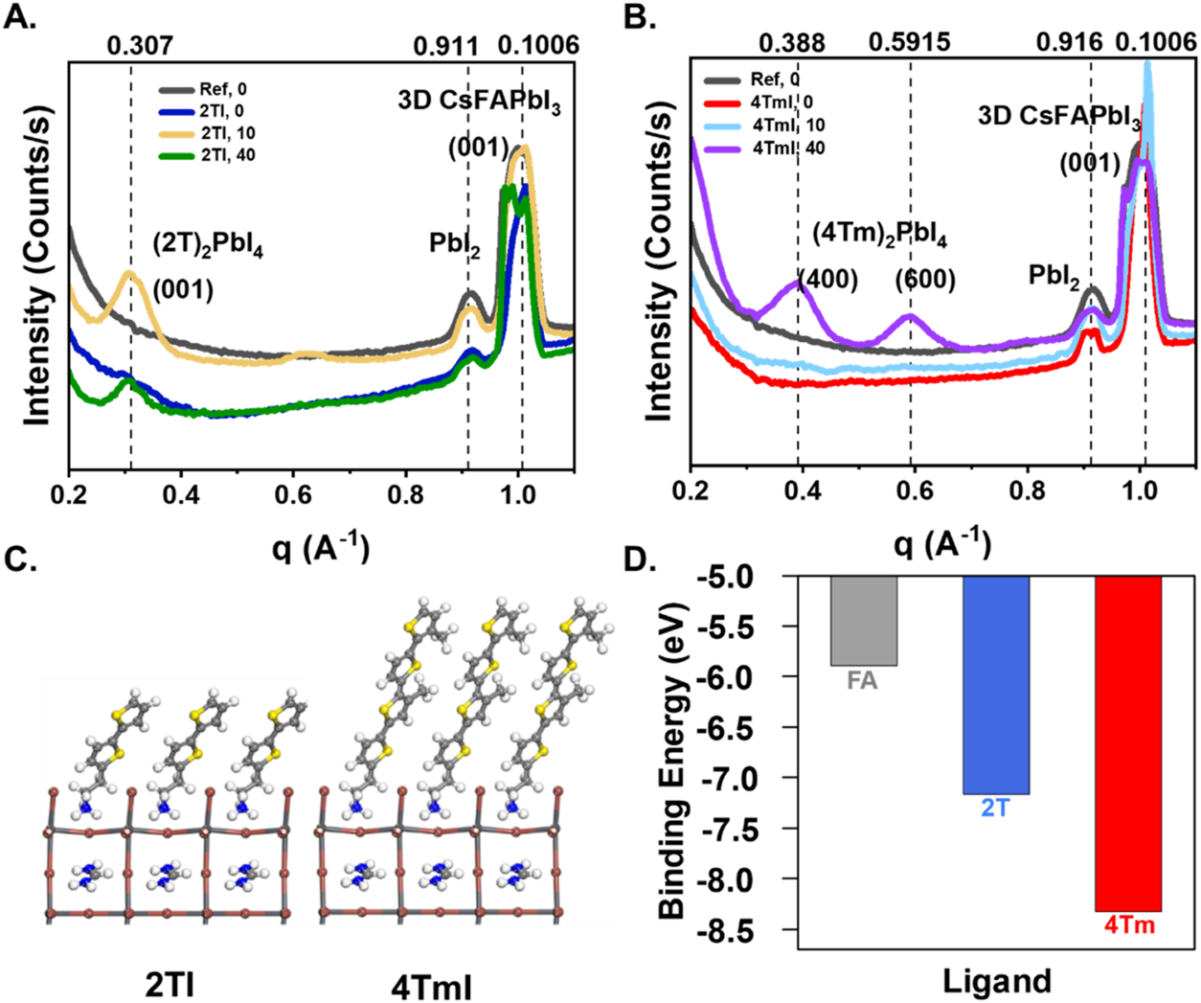
GIWAXS intensity profiles (sector averaged along ±20° from the vertical *q*_*z*_ of the 2D plots) comparing reference perovskite with (A) 2TI and (B) 4TmI passivated films. α_incident_ = 0.1° (C) Anchoring configuration and (D) binding energy of different cations to the [PbI_6_]^4–^ sublattice.

Since we prepared our CsFAPbI_3_ films with PbI_2_ excess, we also tracked the evolution of the PbI_2_ peak during annealing, hypothesizing that excess PbI_2_ will react with the passivation cations and take part in phase conversion. Peak areas and the full width at half-maxima (FWHM) calculated after background subtraction are summarized in Table S3. Introducing passivation molecules reduces the peak area associated with excess PbI_2_ present on the perovskite surface (as observed in Ref, 0 films). During annealing, we observed a temporary increase in the PbI_2_ area without significant peak broadening at the 10 min mark in both molecules. This transient spike occurs during the surface reconstruction of 3D perovskite, as the cations react with excess PbI_2_ to form a two-dimensional perovskite layer. Additionally, increase in PbI_2_ content with annealing time has been reported in another study, which could also be a contributing factor for the observed increase in area.^48^ As the 2D layer forms, PbI_2_ is consumed in the transformation process. Thus, after 40 min, we observe a reduction in peak area. In 4TmI, 40, this is accompanied by FWHM broadening, indicating a reduced crystallite size for the remaining PbI_2_. The fact that we still record finite peaks after 40 min of annealing suggests that not all excess PbI_2_ is incorporated in the 3D to 2D restructuring, and that residual PbI_2_ is present atop the passivation layer.

To understand the strength of the interaction of the 2T^+^ and 4Tm^+^ cations with the perovskite surface, we performed DFT calculations. Our results, presented in Figure 2C,D, suggest strong anchoring of 4Tm^+^ cations atop the inorganic [PbX_6_]^4–^ sublattice, as indicated by a higher (negative) binding energy than both 2T^+^ and FA^+^. As both cations rely on the same anchoring group, the distinguishing factor lies in the degree of π conjugation. For 4TmI, increased π–π interactions between the molecules serve to stabilize a monolayer of molecules on the surface of the perovskite once the monolayer is formed.

### 2.2 Proposed Mechanism

Based on our findings, we present a model describing the time-dependent thermal diffusion behavior for the two molecules in Figure 3. PbI_2_ peak evolution observed from GIWAXS suggests a dynamic interaction between the passivation molecules and excess PbI_2_ on the perovskite surface. As seen from the transient PbI_2_ peak area changes after 10 min of annealing for both cations, PbI_2_ participates in the 3D to 2D structural reorganization of perovskites by reacting with 2TI/4TmI to form the *n* = 1 Ruddlesden–Popper phase. For the 2TI-treated perovskite film, this phase conversion is achieved within the first 10 min of annealing. On prolonged annealing for 40 min, loss in crystallinity of the interface is observed based on our GIWAXS patterns. On the other hand, 4TmI, on account of its larger size, exhibits slower diffusion and reaction kinetics, resulting in the delayed formation of the *n* = 1 phase, which becomes apparent only after 40 min annealing.

**Figure 3:**
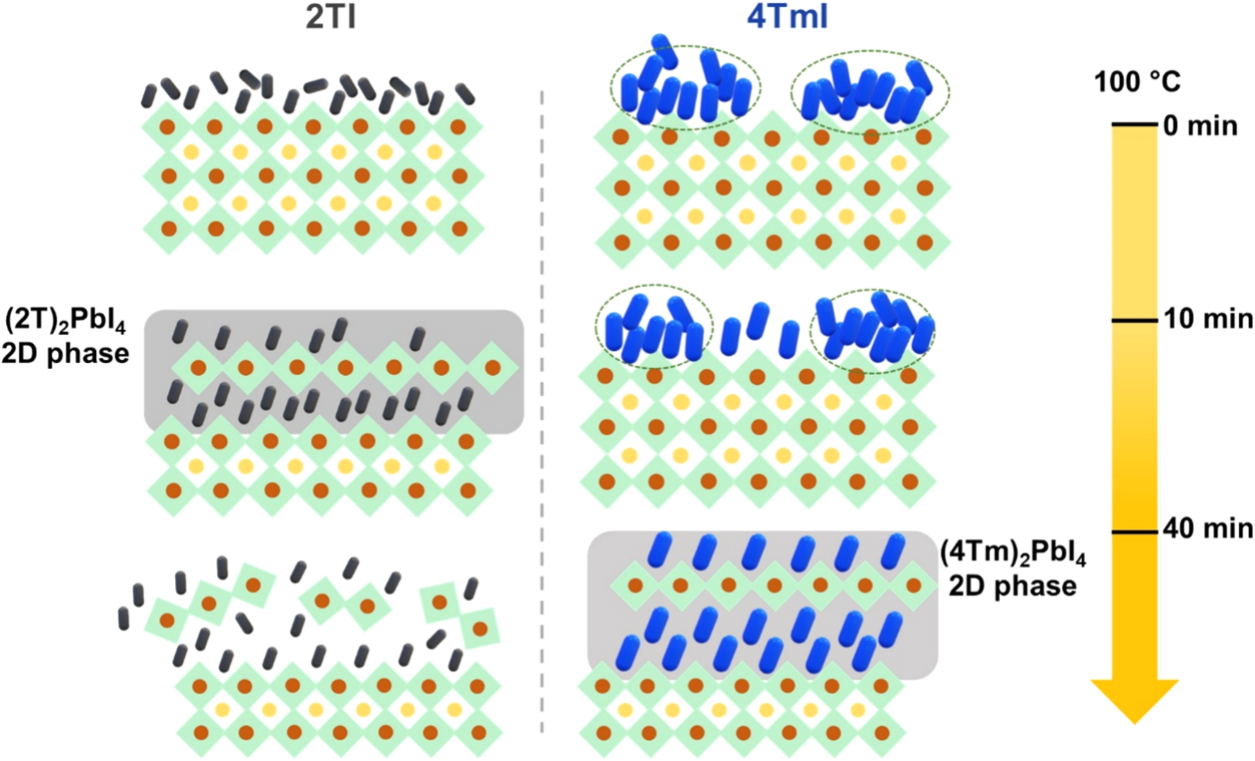
Schematics representing the proposed evolution of the surface layer of perovskite films treated with 2TI and 4TmI salts during annealing.

### 2.3 Device Optoelectronic Properties after Surface Treatment

To understand how the observed interfacial changes affect device performance, we fabricated solar cells with and without a surface treatment with the bulky cations, using the n-i-p configuration shown in Figure 4A. Figure 4B–D presents box plots of stabilized PCE (PCE after 1 min of maximum power point tracking), open-circuit voltage (*V*_OC_), and short-circuit current (*J*_SC_) for both passivated and pristine perovskite devices. Devices for each variation were studied before and after annealing at 100 °C for 40 min to understand the long-term impact of perovskite surface modifications on *V*_OC_ and charge extraction. Note that the reported annealing time is in addition to the perovskite layer annealing time, which is 20 min at 150 °C. A complete summary of the parameters obtained from the *J*–*V* scans of the solar cells is presented in Table S1.

**Figure 4:**
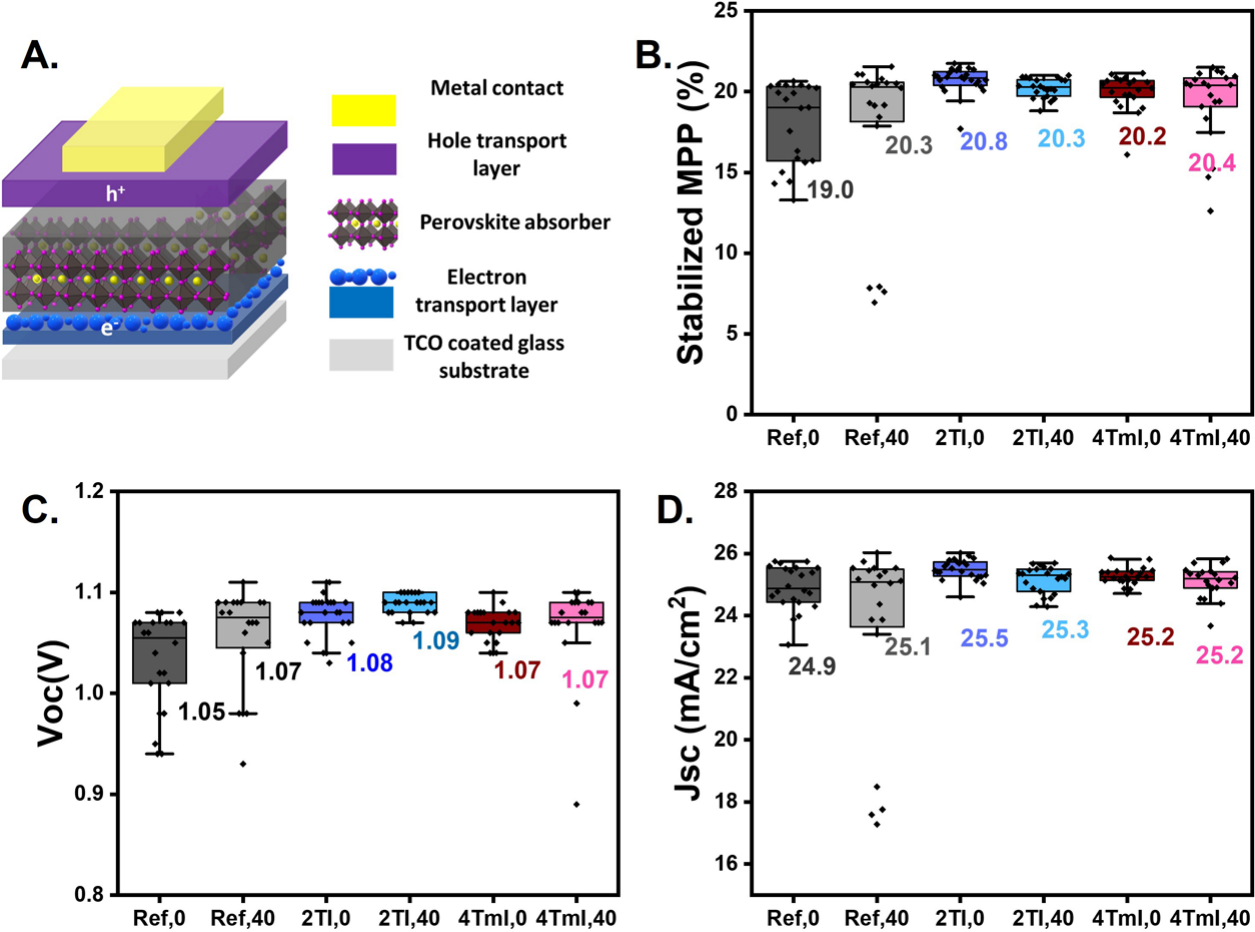
Statistics of device performance for perovskite solar cells with different passivation layers: (A) device schematic, (B) stabilized PCE at the maximum power point, (C) *V*_OC_, and (D) *J*_SC_. Device parameters are obtained from reverse *J*–*V* scans, and their median values are shown beside the boxes.

The reference device (Ref, 0) exhibits a median stabilized PCE of 19.0% during maximum power point (MPP) tracking, with a median *V*_OC_ of 1.05 V and a *J*_SC_ of 24.9 mA cm^–2^. Upon introducing the 2TI passivation layer, the device demonstrates a notable improvement in performance, achieving a median stabilized PCE of 20.8%, *V*_OC_ of 1.08 V, and *J*_SC_ of 25.5 mA cm^–2^. These enhancements suggest an effective reduction in interfacial defect density, leading to reduced recombination and improved charge extraction through the interface.^19,49^ In contrast, the 4TmI passivation layer shows a reduced improvement, with a median stabilized PCE of 20.2%, *V*_OC_ of 1.07 V, and *J*_SC_ of 25.2 mA cm^–2^. The distribution of performances highlights greater variability in the reference device compared with the passivated devices, as evidenced by the broader range of PCE values in Figure 4B. In contrast, passivated devices exhibit tighter distributions, reflecting more consistent device performance.

After thermal stressing, the stabilized PCE for 2TI passivated devices shows minimal loss (20.8% before versus 20.3% after annealing), while that for 4TmI passivated devices shows a slight increase (20.2% before versus 20.4% after annealing). The limited change in performance under thermal stress demonstrated by 2TI and 4TmI contrasts with previous studies using cations like PEAI, highlighting the promise of these molecules for enabling thermally stable perovskite photovoltaics with interface passivation.^27^ For the annealed 2TI surface treatment, slight reductions in *J*_SC_ suggest reduced charge extraction. Based on the structural data discussed above, this could be ascribed to the formation of a 2D insulating spacer layer after 40 min of annealing. Notably, the 2D spacer layer formed following 4TmI surface treatment does not seem to adversely affect charge extraction, as evidenced by the stable J_SC_ values in annealed 4TmI devices. Additionally, nonradiative recombination behavior appears to remain unaffected, as indicated by the improved *V*_OC_ values for 2TI and the consistent values for 4TmI-treated devices.

Among all of our devices, the champion device was obtained by passivating perovskite with the 2TI surface treatment layer without subjecting it to any thermal stress, with a maximum reverse scan PCE of 22.3% (Table S1), a *V*_OC_ of 1.1 V, a *J*_SC_ of 26.02 mA cm^–2^, and a fill factor (FF) of 79.21% obtained from a reverse *J*–*V* scan. A maximum stabilized PCE of 21.7% is obtained on maximum power point tracking.

To rationalize the stable PCE of 4TmI-treated solar cells, we refer to the literature on the cation, which reports lowering of the hole extraction barrier at the perovskite/HTL interface due to better alignment of valence band maxima (VBM) with the highest occupied molecular orbitals (HOMO) in Spiro-OMeTAD.^45^ This favorable band alignment could explain the fact that performances do not suffer despite the formation of a 2D (4Tm)_2_PbI_4_ spacer layer after thermal treatment (comparing 4TmI, 0 and 4TmI, 40).

On the other hand, the electronic structure of the 2TI/perovskite system has not been extensively studied. Band alignments reported for 2D (2T)_2_PbI_4_ and (4Tm)_2_PbI_4_ heterostructures in the literature show that the former has a deeper HOMO position (of the organic 2T^+^ organic layer) and larger bandgap compared to the latter (see Figure S9 for a proposed energy-level alignment).^34,44^ This makes the preservation of the device performance in 2TI-treated films less straightforward to explain. The preserved PCEs could arise from the combined effects of cation-induced energy-level shifts and surface passivation effects, but to say so conclusively requires a more in-depth characterization of the electronic properties of the passivated perovskite thin films, which will be an object of future study.

## 3 Conclusions

In this work, we demonstrated that the design of conjugated passivation molecules controls the rate of reconstruction of the surface layer of the 3D perovskite. This is evidenced by the faster formation of a 2D surface layer in 2TI passivated films compared to the larger 4TmI. Efficient charge extraction can be maintained despite the transition to their 2D phases, allowing the passivated devices to perform well under thermal stress with minimal PCE loss. Our findings suggest that interfacial phase conversion is not inherently detrimental to device performance, as has been observed in commonly used passivation molecules such as PEAI. By engineering molecular design of interfacial passivation agents, large, conjugated molecules can help in improving the thermal stability of lead halide perovskite solar cells. At the same time, our study highlights the need to focus on other factors, such as interfacial electronic band alignment and surface energy, which may be responsible for retaining charge extraction and preserving PCEs.

## 4 Experimental Section

### 4.1 Materials

To prepare the Cs_0.05_FA_0.95_PbI_3_ perovskite solution, a 1.5 M solution of PbI_2_ (Tokyo Chemical Industry, TCI) was prepared in 4:1 DMF/DMSO (ratio by volume, Sigma-Aldrich). The PbI_2_ stock solution was then stirred and heated to 80 °C for 45 min. Upon cooling, the PbI_2_ precursor solution was added to 0.22 g of formamidinium (FAI, Dynamo) powder to prepare a FAPbI_3_ stock, with a 1.05:1 (PbI_2_:FAI) molar ratio. 13.23 μL of 4:1 DMF:DMSO was further added to the solution to obtain 1.24 M FAPbI_3_. Finally, the FAPbI_3_ solution was mixed with 1.5 M CsI (Sigma-Aldrich) dissolved in DMSO to obtain a 95:5 ratio by volume. The solution was prepared in a nitrogen glovebox.

### 4.2 Device Fabrication

Solar cells with direct n–i–p architecture were prepared on 1 in. × 1 in. patterned FTO glass substrate using a solution-based method. The substrates were sequentially sonicated in a 2% Hellmanex solution, DI water, acetone (99.5% pure, Sigma-Aldrich), and IPA (99.5% pure, Sigma-Aldrich) for 20 min each, followed by drying with a nitrogen gun and placing on a hot plate. Glass slides were used to cover the edges of the substrates, leaving the active area exposed. An electron transport layer (ETL) consisting of a compact layer of TiO_2_ was then deposited at 450 °C through spray pyrolysis with a 3.5 L min^–1^ flow of oxygen carrier gas. The compact TiO_2_ solution was prepared by mixing 0.480 mL of acetylacetone (Sigma-Aldrich) and 0.720 mL of titanium diisopropoxide bis­(acetylacetonate) (75 wt % in 2-propanol, Sigma-Aldrich) diluted in 10.8 mL of ethanol (99.9% pure, anhydrous, Sigma-Aldrich). The solution was sprayed using a Sparmax spray gun in 10–15 s back and forth passages, with a 30 s delay between cycles, which continued until the entire solution was utilized. The substrates were annealed at 450 °C for an additional 30 min before being allowed to cool to room temperature. This was followed by spin coating 60 μL of mesoporous TiO_2_ solution prepared by dissolving 150 mg mL^–1^ titanium dioxide paste (Sigma-Aldrich) in ethanol (99.9% pure, anhydrous, Sigma-Aldrich), at 4000 rpm for 10 s, followed by annealing at 100 °C for 20 min. The substrate’s edges during spin coating were covered with magic tape to prevent TiO_2_ deposition. The substrates were then sintered at a temperature starting at 450 °C and ramping down to 150 °C, before being transferred to a glovebox with <10 ppm of O_2_ and H_2_O. On top of the ETL, 60 μL of 1.24 M Cs_0.05_FA_0.95_PbI_3_ perovskite solution was spin-coated at 1000 rpm for 10 s, followed by 6000 rpm for 20 s. 250 μL of chlorobenzene (99.5% pure, Sigma-Aldrich) was dropped at 26 s from the start of the spin coating recipe. The devices were then annealed at 150 °C for 20 min. For top passivation, 80 μL each of either 1.35 mg mL^–1^ 2TI or 2.12 mg mL^–1^ 4TmI dissolved in isopropyl alcohol (99.5%, anhydrous, Sigma-Aldrich) was dynamically spin-coated on top of the perovskite film at 5000 rpm, 5000 rpm/s, and 20 s. The films were then annealed at 100 °C for either 0, 10, or 40 min. For depositing the hole transport layer (HTL), a 0.07 M solution of Spiro-OMeTAD powder (1 M) dissolved in chlorobenzene­(anhydrous, Sigma-Aldrich) was mixed with 67.06 μL of 4-*tert*-butylpyridine (98% pure, Sigma-Aldrich), 30 μL of 1.8 M Li-TFSI (in acetonitrile stock solution, Sigma-Aldrich), and 16.64 μL of 0.25 M Co­(II) salt (FK209—in acetonitrile stock solution, 98% pure, Sigma-Aldrich), in that order. 90 μL of this solution was dynamically spin-coated, dispensing at 1–2 s after the start of spin coating at 3000 rpm, 3000 rpm/s for 30 s. The CsFAPbI_3_ and Spiro-OMeTAD films were cleaned off the substrate edges using dimethylformamide (ACS reagent, ≥ 99.8%, Acros Organics) and then acetonitrile (ACS reagent, ≥99.5%, Sigma-Aldrich). Finally, a 43 nm thick Au top gold electrode was thermally evaporated on top of the HTL to define a 0.128 cm^–2^ active area.

### 4.3 Film Characterization

Scanning electron microscopy (SEM) images of passivated perovskite films on FTO glass substrates (i.e., FTO/perovskite/2TI and FTO/perovskite/4TmI) were obtained with a Hitachi SU8230 secondary electron detector to determine the film morphology at an accelerating voltage of 3.5 kV and current of 10 μA. Carbon tape was used to connect all film samples to the sample stub to ensure good electrical conductivity and thus well-resolved images. XPS for elemental composition analysis was performed on perovskite films deposited on the FTO substrates. Survey scans were obtained with a Thermo Scientific K α X-ray photoelectron spectrometer using Cu Kα. Scan parameters were 200 eV pass energy and 1 eV energy step; elemental scan parameters were 50 eV pass energy and 0.1 eV energy step. Profiles were obtained for Pb 4f (5 scans), I 3d (5 scans), C 1s (20 scans), N 1s (10 scans), and S 2p (10 scans). Thermo Scientific Avantage software was used to perform surface analysis from the data; the Pb-X peak position was used as the reference to perform a binding energy shift for all samples. To perform grazing incidence wide-angle X-ray scattering (GIWAXS) measurements on perovskite/passivation films deposited on FTO glass substrates, images were taken on 0.5 × 0.5 sized samples at beamline 11-BM at National Synchrotron Light Source II at Brookhaven National Laboratory. An X-ray beam with energy 13.5 keV was used, with 1 mrad divergence, beam width of 0.2 mm, height of 0.05 mm, and 0.7% resolution, to obtain images at incidence angles of 0.05, 0.1, and 0.5° with an exposure time of 20 s. SciAnalysis software package was used for data analysis. AFM topography and KPFM measurements were performed by using an Asylum Cypher. All KPFM images were acquired using the same cantilever, an asyelec-01-R2: lever Ti/Ir (5/20) and tip Ti/Ir (5/20), *f* = 75 Hz, *k* = 2.8 N m^–1^.

### 4.4 Device Characterization

For measurement of photovoltaic characteristics, a Fluxim Litos Lite setup equipped with Wavelabs Sinus-70 AAA solar simulator with AM1.5 spectrum was used to obtain *J*–*V* curves. Forward and reverse *J*–*V* scans were collected at 30 mV s^–1^ scan rate, and stabilized power output was collected with maximum power point tracking for 60 s. Measurements were performed under nitrogen flow in the chamber over a pixel area of 0.0625 cm^–2^ defined by placing masks over the device.

## Supplementary Material



## Notes

Schematic of spin coating method to deposit perovskite and passivation films; SEM images showing film morphology of reference and passivated samples; KPFM mapping of contact potential difference on the surface of reference and passivated samples; XPS elemental scans for both 2TI- and 4TmI-treated perovskite films; 2D GIWAXS profiles showing crystal structure of 2TI- and 4TmI-treated perovskite films at surface and in bulk; intensity profiles showing crystal structure of 2TI- and 4TmI-treated perovskite films in bulk; proposed energy-level alignments for (2T)­2PbI_4_ and (4Tm)­2PbI_4_ 2D perovskites; summary of device performances of reference and passivated perovskite solar cells; calculation of I/Pb and N/Pb elemental ratios derived from XPS; and calculation of PbI_2_ peak areas and fwhm derived from GIWAXS intensity profiles. (PDF)

## References

[1] He C., Liu X. (2023). The rise of halide
perovskite semiconductors. Light: Sci. Appl..

[2] Akkerman Q. A., Manna L. (2020). What Defines a Halide
Perovskite. ACS Energy
Lett..

[3] Manser J. S., Christians J. A., Kamat P. V. (2016). Intriguing Optoelectronic Properties
of Metal Halide Perovskites. Chem. Rev..

[4] Colella S., Mosconi E., Fedeli P. (2013). MAPbI3-xClx Mixed Halide
Perovskite for Hybrid Solar Cells: The Role of Chloride as Dopant
on the Transport and Structural Properties. Chem. Mater..

[5] NREL Photovoltaic Research . Best Research-Cell Efficiency Chart. https://www2.nrel.gov/pv/cell-efficiency (accessed April 22, 2025).

[6] Xia J., Liang C., Gu H. (2023). Surface Passivation
Toward Efficient and Stable Perovskite Solar Cells. Energy Environ. Mater..

[7] Ma K., Sun J., Atapattu H. R. (2023). Holistic energy landscape management in
2D/3D heterojunction via molecular engineering for efficient perovskite
solar cells. Sci. Adv..

[8] Liu G., Zheng H., Xu H. (2020). Interface passivation
treatment by halogenated low-dimensional perovskites for high-performance
and stable perovskite photovoltaics. Nano Energy.

[9] Sherkar T. S., Momblona C., Gil-Escrig L., Bolink H. J., Koster L. J. A. (2017). Improving
Perovskite Solar Cells: Insights From a Validated Device Model. Adv. Energy Mater..

[10] Perini C. A. R., Doherty T. A. S., Stranks S. D., Correa-Baena J.-P., Hoye R. L. Z. (2021). Pressing challenges in halide perovskite
photovoltaics-
from the atomic to module level. Joule.

[11] Stolterfoht M., Caprioglio P., Wolff C. M. (2019). The impact of energy
alignment and interfacial recombination on the internal and external
open-circuit voltage of perovskite solar cells. Energy Environ. Sci..

[12] Zhang Q., Xiong S., Ali J. (2020). Polymer interface engineering
enabling high-performance perovskite solar cells with improved fill
factors of over 82%. J. Mater. Chem. C.

[13] Lee S. H., Hong S., Lee H. H., Kim H. J. (2021). Interface Engineering
of Perovskite/Hole Transport Layer Using Nano-Network Formation in
Small Molecule–Polymer Blend for Efficient Inverted Perovskite
Solar Cells. Adv. Mater. Interfaces.

[14] Bi H., Guo Y., Guo M. (2023). Reduced interfacial recombination losses and
lead leakage in lead-based perovskite solar cells using 2D/3D perovskite
engineering. J. Power Sources.

[15] Liu G., Zheng H., Ye J. (2021). Mixed-Phase Low-Dimensional
Perovskite-Assisted Interfacial Lead Directional Management for Stable
Perovskite Solar Cells with Efficiency over 24%. ACS Energy Lett..

[16] Abate A., Saliba M., Hollman D. J. (2014). Supramolecular
Halogen
Bond Passivation of Organic–Inorganic Halide Perovskite Solar
Cells. Nano Lett..

[17] Noel N. K., Abate A., Stranks S. D. (2014). Enhanced Photoluminescence
and Solar Cell Performance via Lewis Base Passivation of Organic–Inorganic
Lead Halide Perovskites. ACS Nano.

[18] Li J., Bu T., Lin Z. (2021). Efficient and stable perovskite solar cells
via surface passivation of an ultrathin hydrophobic organic molecular
layer. Chem. Eng. J..

[19] Sherkar T. S., Momblona C., Gil-Escrig L. (2017). Recombination in Perovskite
Solar Cells: Significance of Grain Boundaries, Interface Traps, and
Defect Ions. ACS Energy Lett..

[20] Guo Y., Apergi S., Li N. (2021). Phenylalkylammonium
passivation enables perovskite light emitting diodes with record high-radiance
operational lifetime: the chain length matters. Nat. Commun..

[21] Wu T., Wang Y., Dai Z. (2019). Efficient and Stable
CsPbI3 Solar Cells via Regulating Lattice Distortion with Surface
Organic Terminal Groups. Adv. Mater..

[22] Zhang J., Duan J., Zhang Q. (2022). Understanding
steric-charge-dependence
of conjugated passivators on π-Pb2+ bond strength for efficient
all-inorganic perovskite solar cells. Chem.
Eng. J..

[23] Miao Y., Chen Y., Chen H., Wang X., Zhao Y. (2021). Using steric
hindrance to manipulate and stabilize metal halide perovskites for
optoelectronics. Chem. Sci..

[24] Nakamura M., Takenaka I., Mabuchi T. (2022). Thermal Stability of
K-Doped Organometal Halide Perovskite for Photovoltaic Materials. ACS Appl. Energy Mater..

[25] Hoye R. (2021). The Role of Dimensionality on the Optoelectronic Properties of Oxide
and Halide Perovskites, and their Halide Derivatives. Adv. Energy Mater..

[26] Lee H. B., Kumar N., Tyagi B. (2021). Bulky organic cations
engineered lead-halide perovskites: a review on dimensionality and
optoelectronic applications. Mater. Today Energy.

[27] Perini C. A. R. (2022). Interface Reconstruction
from Ruddlesden–Popper
Structures Impacts Stability in Lead Halide Perovskite Solar Cells. Adv. Mater..

[28] Song J., Liu H., Pu W. (2022). Thermal instability originating from the interface
between organic–inorganic hybrid perovskites and oxide electron
transport layers. Energy Environ. Sci..

[29] Qiu J., Zheng Y., Xia Y. (2019). Rapid Crystallization
for Efficient 2D Ruddlesden–Popper (2DRP) Perovskite Solar
Cells. Adv. Funct. Mater..

[30] Ma C., Shen D., Ng T.-W., Lo M.-F., Lee C.-S. (2018). 2D Perovskites
with Short Interlayer Distance for High-Performance Solar Cell Application. Adv. Mater..

[31] Cheng Y., Wan H., Sargent E. H., Ma D. (2024). Reduced-Dimensional Perovskites:
Quantum Well Thickness Distribution and Optoelectronic Properties. Adv. Mater..

[32] Katan C., Mercier N., Even J. (2019). Quantum and Dielectric
Confinement
Effects in Lower-Dimensional Hybrid Perovskite Semiconductors. Chem. Rev..

[33] Sun K., Guo R., Liu S. (2024). Deciphering Structure
and Charge Carrier Behavior
in Reduced-Dimensional Perovskites. Adv. Funct.
Mater..

[34] Gao Y., Shi E., Deng S. (2019). Molecular engineering
of organic–inorganic
hybrid perovskites quantum wells. Nat. Chem..

[35] Mahal E., Mandal S. C., Pathak B. (2022). Understanding
the role of spacer
cation in 2D layered halide perovskites to achieve stable perovskite
solar cells. Mater. Adv..

[36] Zhang W., Yuan M., Han L. (2023). Perovskite Surface Passivation
Using Thiophene-Based Small Molecules for Efficient and Stable Solar
Cells. ACS Appl. Energy Mater..

[37] Fu Y., Li Y., Xing G., Cao D. (2022). Surface passivation of perovskite
with organic hole transport materials for highly efficient and stable
perovskite solar cells. Mater. Today Adv..

[38] Ni C., Huang Y., Zeng T. (2020). Thiophene Cation Intercalation
to Improve Band-Edge Integrity in Reduced-Dimensional Perovskites. Angew. Chem., Int. Ed..

[39] Gunes U., Bag Celik E., Akgul C. C. (2021). A Thienothiophene-Based
Cation Treatment Allows Semitransparent Perovskite Solar Cells with
Improved Efficiency and Stability. Adv. Funct.
Mater..

[40] Ma Y., Zhang L., Xu Y. (2022). Internal Interactions
between Mixed Bulky Organic Cations on Passivating Defects in Perovskite
Solar Cells. ACS Appl. Mater. Interfaces.

[41] Chen L., Chen J., Wang C. (2021). High-Light-Tolerance
PbI2 Boosting the Stability and Efficiency of Perovskite Solar Cells. ACS Appl. Mater. Interfaces.

[42] He Z., Zhou Y., Xu C. (2021). Mechanism of Enhancement
in Perovskite Solar Cells by Organosulfur Amine Constructed 2D/3D
Heterojunctions. J. Phys. Chem. C.

[43] Kundar M., Bhandari S., Chung S. (2023). Surface Passivation
by Sulfur-Based 2D (TEA)(2)­PbI(4) for Stable and Efficient Perovskite
Solar Cells. ACS Omega.

[44] Shi E., Yuan B., Shiring S. B. (2020). Two-dimensional halide
perovskite lateral epitaxial heterostructures. Nature.

[45] Ma K., Atapattu H. R., Zhao Q. (2021). Multifunctional Conjugated
Ligand Engineering for Stable and Efficient Perovskite Solar Cells. Adv. Mater..

[46] Zhang J., Yu H. (2021). Reduced energy loss
enabled by thiophene-based interlayers for high
performance and stable perovskite solar cells. J. Mater. Chem. A.

[47] Hidalgo J., Atourki L., Li R. (2023). Bulky cation hinders
undesired secondary phases in FAPbI3 perovskite solar cells. Mater. Today.

[48] Wang H., Ye F., Liang J. (2022). Pre-annealing
treatment for high-efficiency
perovskite solar cells via sequential deposition. Joule.

[49] Kang B., Han Y. J., Hwang S. J. (2024). Grain boundary passivation
by alkylammonium salt for highly stable perovskite solar cells. J. Ind. Eng. Chem..

