# Supplementary material for: Bulky Cation-Modified Interfaces for Thermally Stable Lead Halide Perovskite Solar Cells
Source: Chem Mater. 2025 May 15;37(10):3676–84. doi: 10.1021/acs.chemmater.4c03468 (PMC12120914; doi:10.1021/acs.chemmater.4c03468)
Supplement: Supplementary file 1 [file cm4c03468_si_001.pdf]

Supplementary Information

Bulky Cation Modified Interfaces for Thermally Stable Lead Halide Perovskite Solar Cells

Sakshi Sharma[1], Carlo A.R. Perini[1]*, Courtney Brea[1], Sarah Wieghold[2], Ruipeng Li[3], Letian Dou[4,5], Antonio Facchetti[1,7], Guoxiang Hu[1,6], Juan-Pablo Correa-Baena[1,6]*

[1]School of Materials Science and Engineering, Georgia Institute of Technology, Atlanta, Georgia 30332, USA
[2]Advanced Photon Source, Argonne National Laboratory, Lemont, Illinois 60439, USA
[3]National Synchrotron Light Source II, Brookhaven National Laboratory, Upton, New York 11973, USA
[4]Davidson School of Chemical Engineering, Purdue University, West Lafayette, Indiana 47907, USA
[5]Birck Nanotechnology Center, Purdue University, West Lafayette, Indiana 47907, USA
[6]School of Chemistry and Biochemistry, Georgia Institute of Technology, Atlanta, Georgia 30332, USA
[7]Department of Chemistry, Northwestern University, Evanston, Illinois 60208, USA

Corresponding authors: jpcorrea@gatech.edu, cperini8@gatech.edu

Perovskite film fabrication

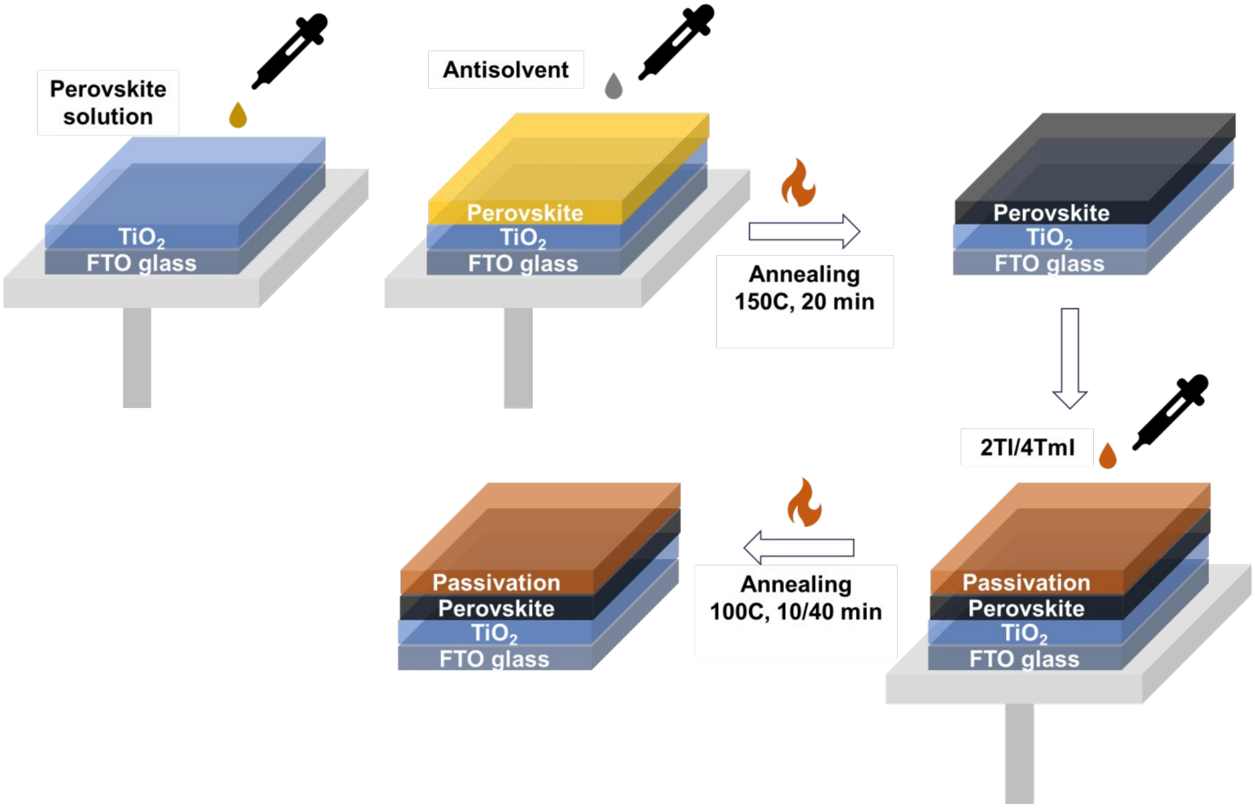

Figure S1. Spin coating method used to deposit perovskite and passivation layers. 4mM solutions of 2TI (1.35mg/mL) and 4TmI (2.12mg/mL) dissolved in isopropyl alcohol were spin coated on $CsFAPbI_3$ thin films.

Film Morphology and Surface Coverage

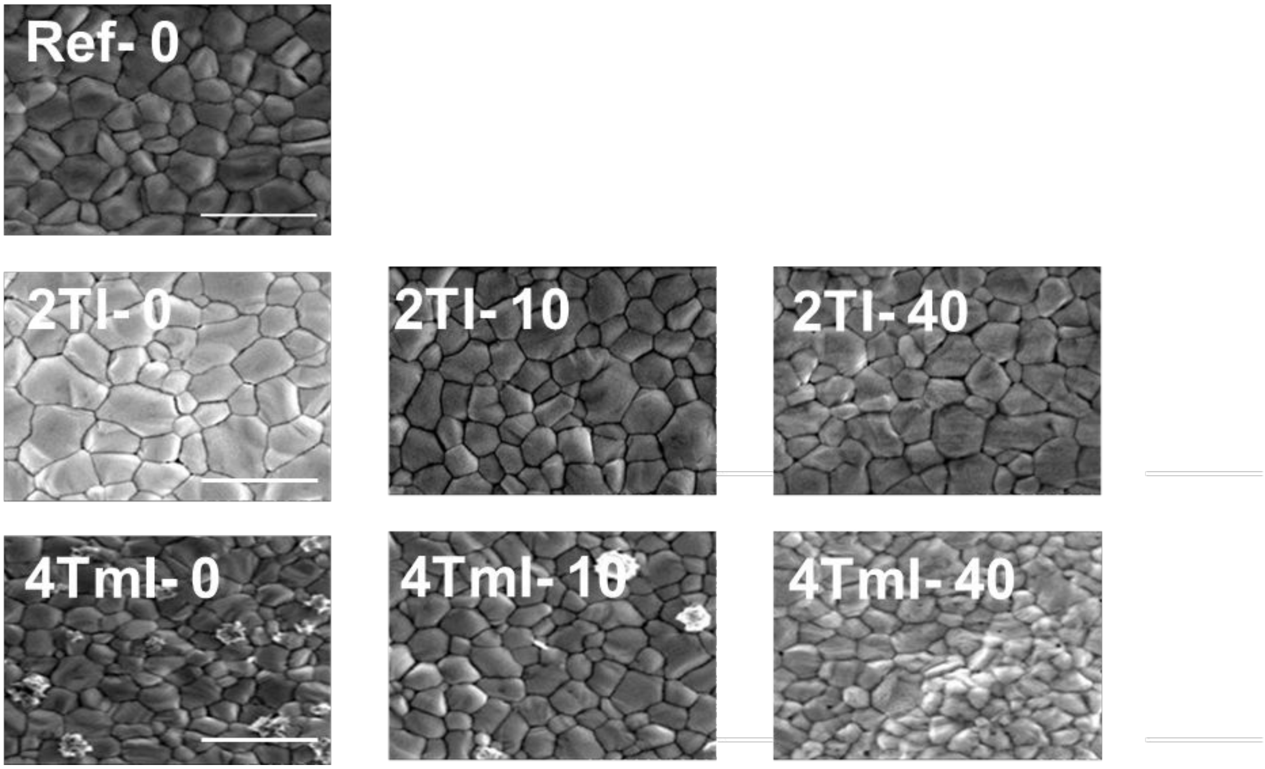

Figure S2. Surface morphology of reference and 2TI/4TmI treated perovskite films (top to bottom) after 0-, 10- and 40-min annealing (left to right). Scale bar is 1μm.

KPFM Surface potential distribution

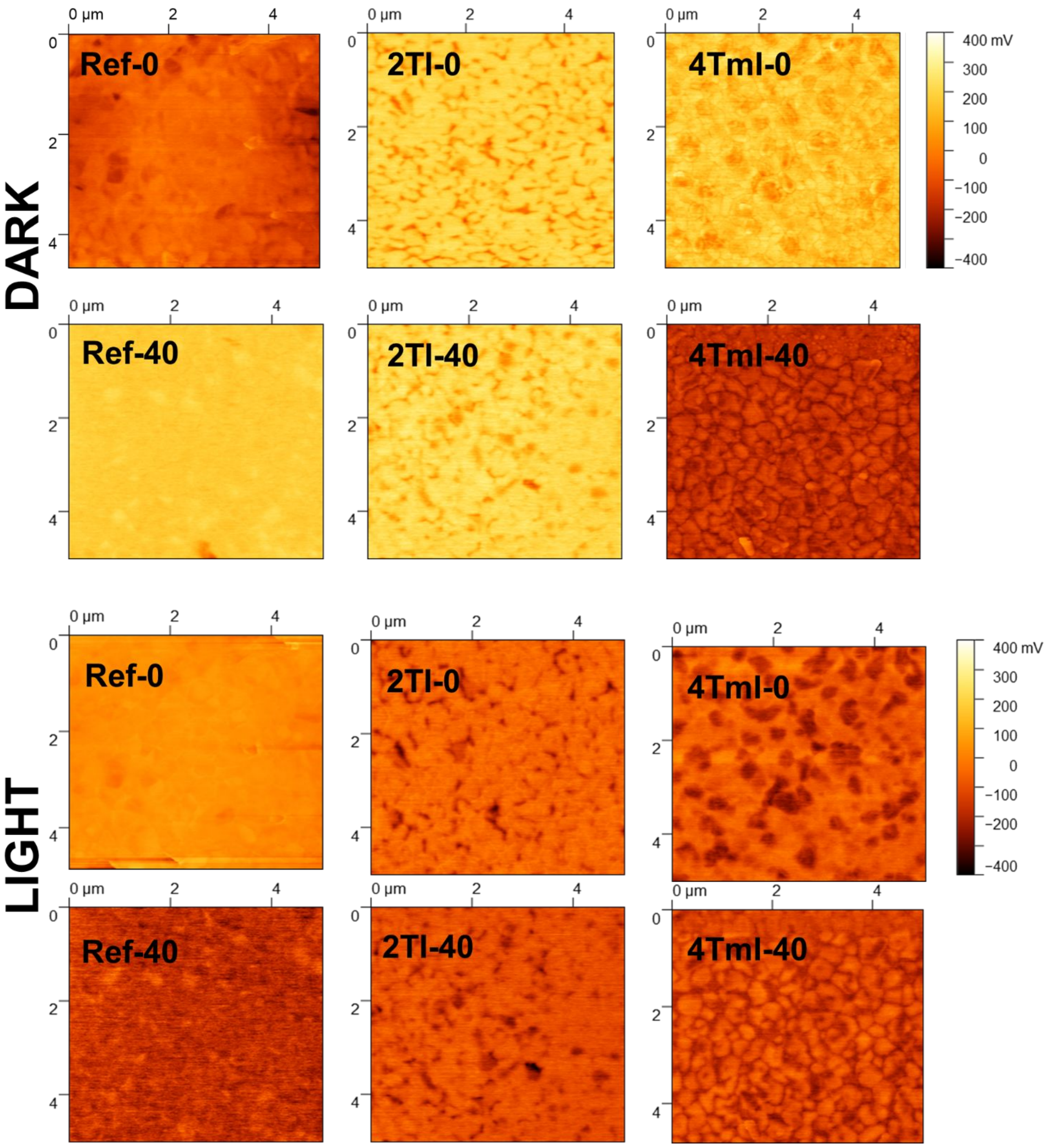

Figure S3. Spatial maps showing Contact Potential Distributions in dark and light. Voltage range for all maps is 0.4V.

Kelvin probe force microscopy (KPFM) was used to measure surface potential of treated films in half stacks of perovskite solar cells without Hole Transport Layer (HTL) and the top electrode.

The Contact Potential Difference (CPD) of the pristine and surface treated films was obtained, in which the electrostatic force between the AFM tip and the sample is minimized[1,2]. The CPD is defined as CPD = Φ_{tip} - Φ_{sample} and is measured at each scan point by applying a DC voltage V_{DC} to compensate for the work function difference. Figure S3 shows that after passivation, the 2TI/4TmI treated films exhibit a heterogeneous voltage response CPD compared to the untreated reference film. This suggests that the interfacial changes upon treatment with the organic layer manifest as spatial contact potential variations.

Notably, we observe from the CPD maps of 4TmI passivated films that the low potential regions (seen as darker spots due to molecular clusters) homogenize on the surface in both the dark and illuminated conditions. This supports our observations from SEM that under prolonged heating of 40 minutes, 4TmI molecules overcome the intermolecular attractive forces that keep them aggregated at the surface and spread over the perovskite film, increasing surface coverage.

Surface Composition Analysis

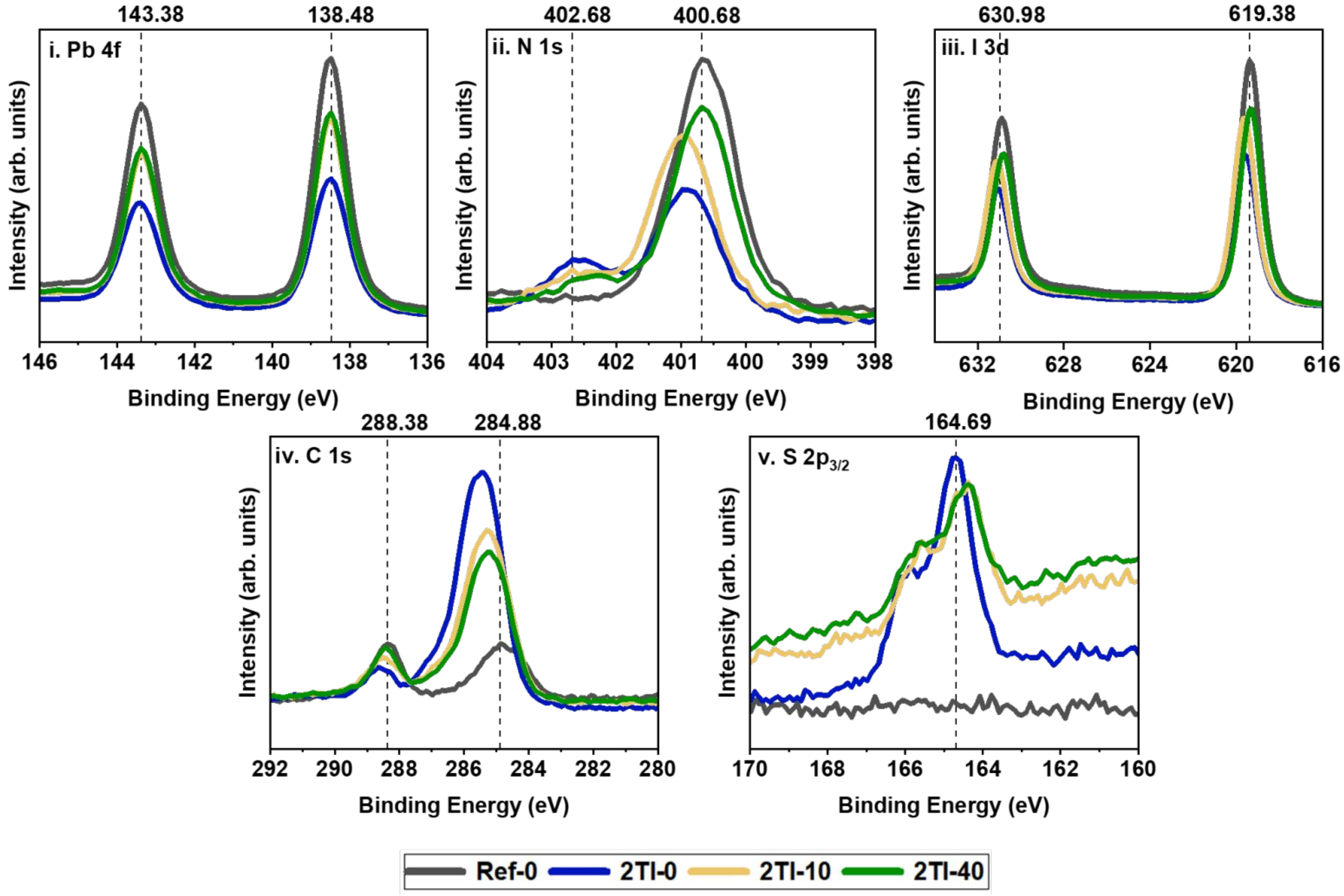

Figure S4. XPS elemental scans for Pb 4f, N 1s, I 3d, C 1s and S $2p_{3/2}$ on the top surface of perovskite films treated with 2TI molecules.

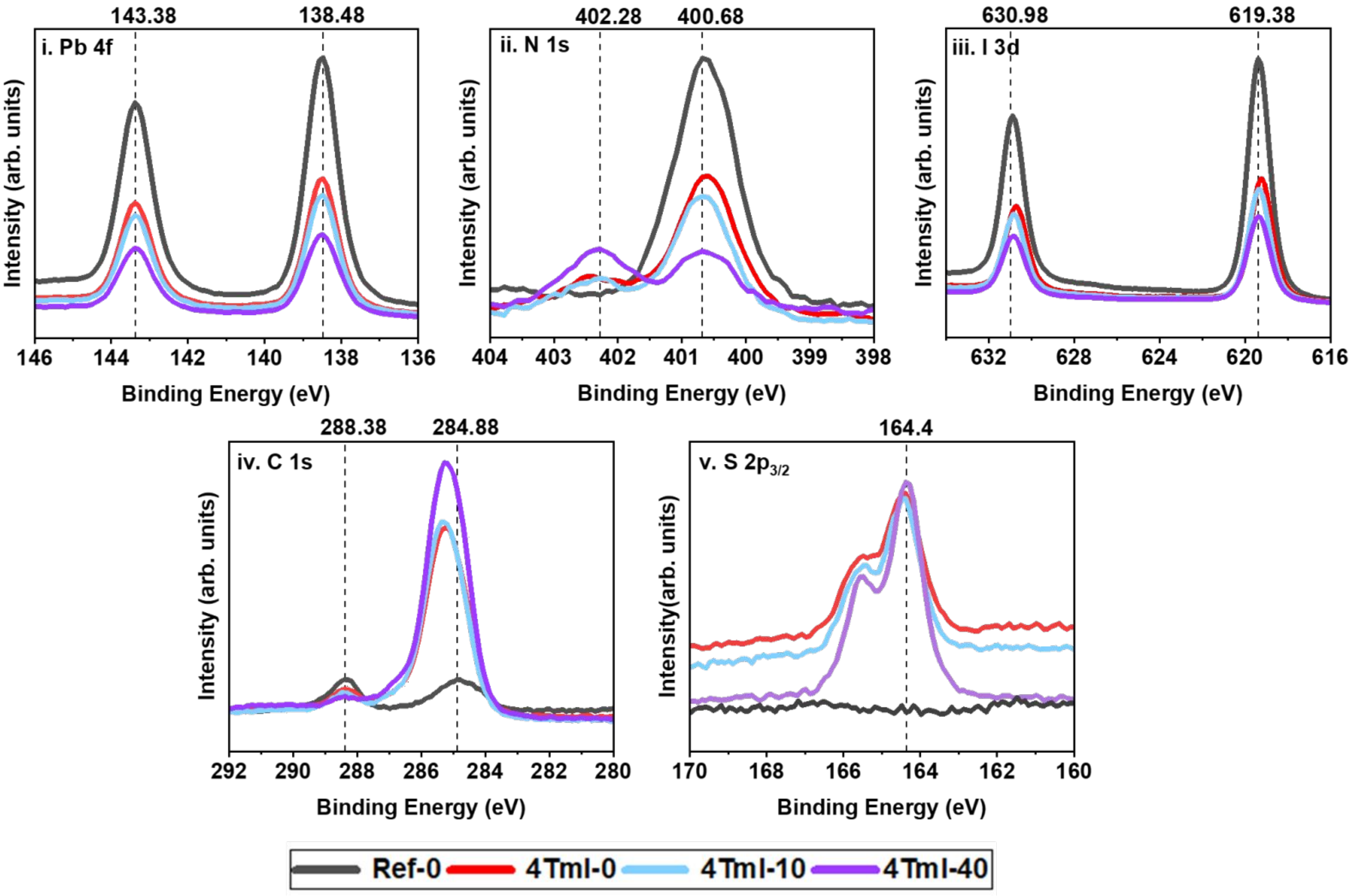

Figure S5. XPS elemental scans for Pb 4f, N 1s, I 3d, C 1s and S $2p_{3/2}$ on the top surface of perovskite films treated with 4TmI molecules.

Surface Structure Analysis

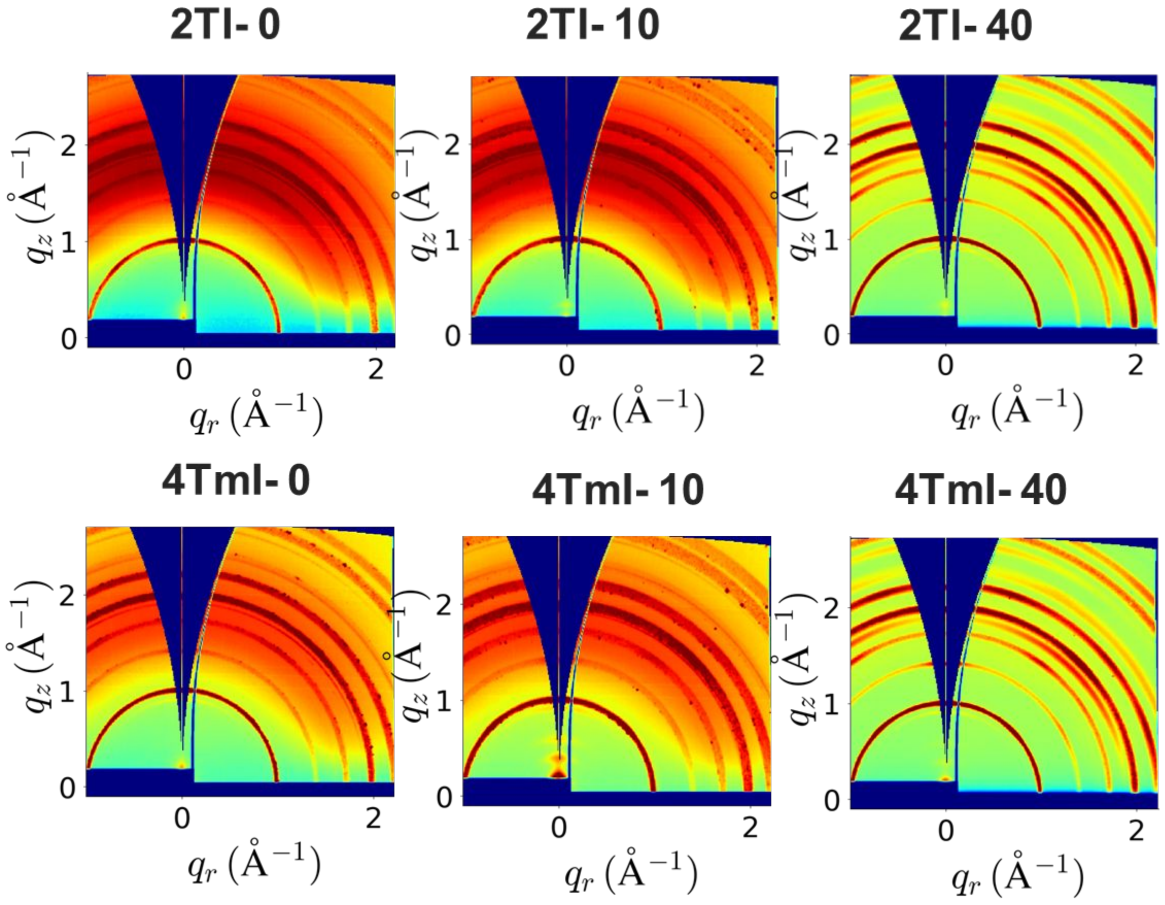

Figure S6. 2D GIWAXS profiles show changes in crystal structure of the interfaces treated with 2TI and 4TmI (top to bottom) after 0-, 10- and 40-min annealing (left to right). For an incident X-ray beam of energy 13.5 keV, $\alpha_{incident}$ = 0.1° corresponds to a depth of around 4 nm (referred to as 'surface').[3]

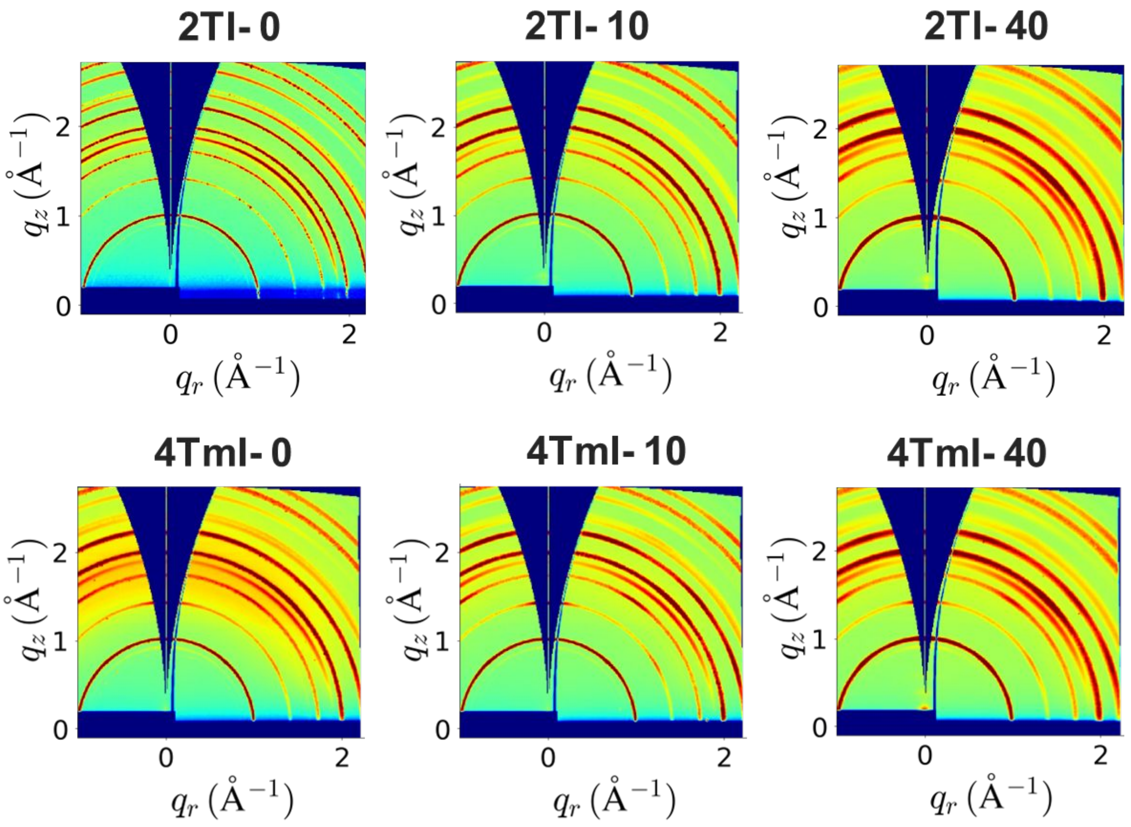

Figure S7. 2D GIWAXS profiles show changes in crystal structure of the interfaces treated with 2TI and 4TmI (top to bottom) after 0-, 10- and 40-min annealing (left to right). For an incident X-ray beam of energy 13.5 keV, $\alpha_{incident}$ = 0.5° corresponds to a depth of around 225 nm (referred to as 'bulk).[3]

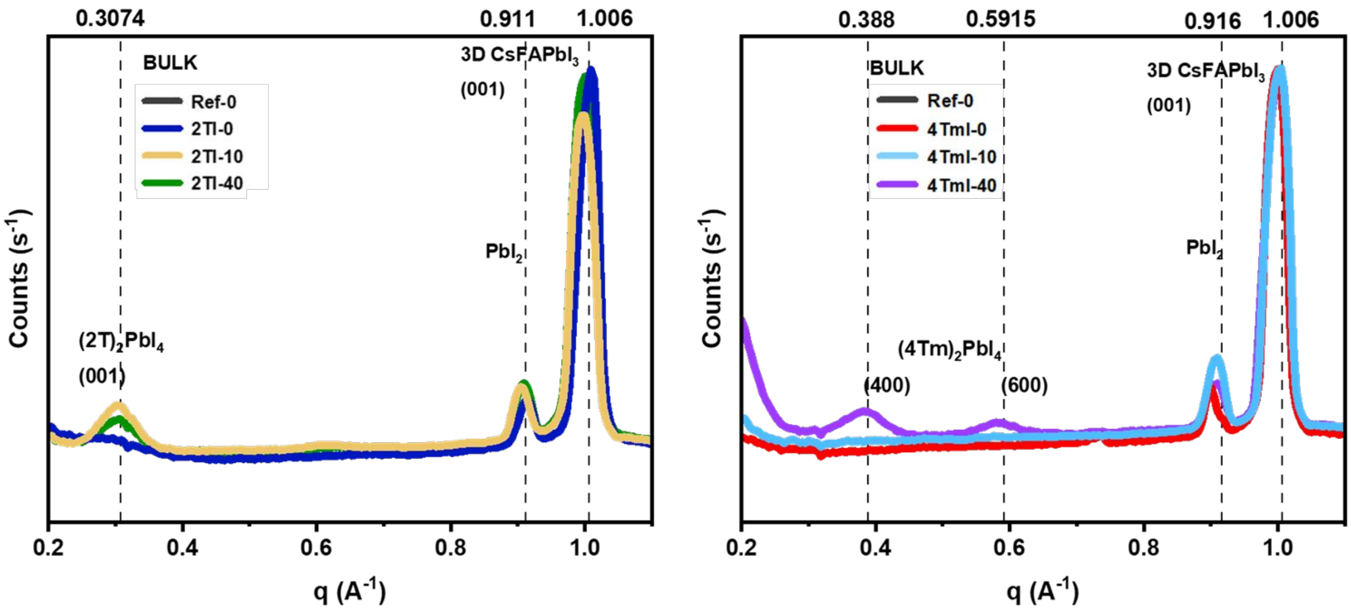

Figure S8. Evolution of the structure of 2TI and 4TmI layers in bulk ($\alpha_{incident} = 0.5°$) with annealing.

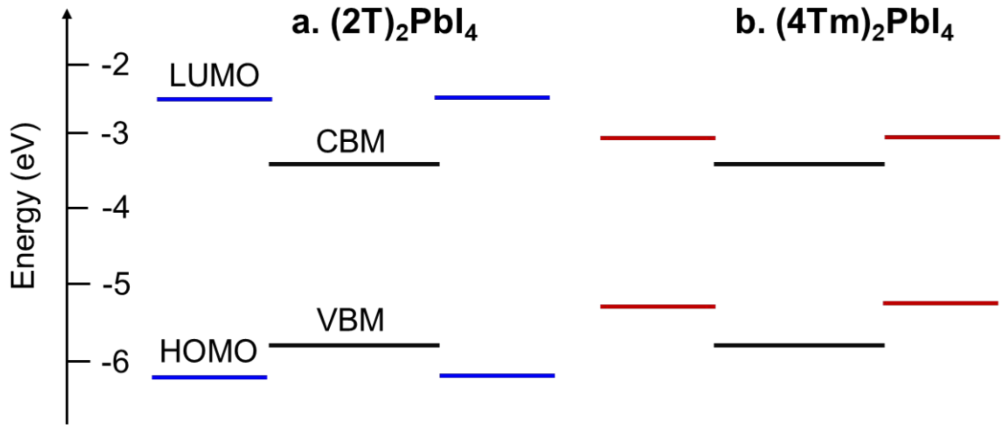

Figure S9. Proposed energy level alignments for (2T)2PbI4 and (4Tm)2PbI4 2D perovskites. The black lines represent Valence Band Maximum (VBM) and Conduction Band Minimum (CBM) for the inorganic layers within the 2D perovskite, whereas the blue and red lines represent the highest occupied molecular orbital (HOMO) and lowest unoccupied molecular orbital (LUMO) of the organic layers within the 2D perovskite.

We have adapted this qualitative band alignment figure for 2D perovskite from literature on 2TI/4TmI molecules. [4,5] Gao et al. measured the energy levels of highest occupied molecular orbital (HOMO) and the lowest unoccupied molecular orbital (LUMO) using a combination of Ultraviolet photoelectron spectroscopy and cyclic voltammetry with computational verification using density functional theory.

Table S1. Summary of photovoltaic performance of reference and 2TI/4TmI treated devices. Median of photovoltaic parameters are extracted from J-V scans in reverse (Rv, bias changing from positive to negative value) and in forward scan directions (Fw, bias changing from negative to positive value). Numbers in parentheses represent the maximum values obtained for each parameter.

Variable	Scan Direction	PCE (%)	V_{oc} (V)	J_{sc} (mA cm^{-2})	FF (%)	Stabilized MPP (%)
**Ref-0**	**Rv**	19.4 (20.8)	1.05 (1.08)	24.9 (25.7)	74.0 (75.6)	19.0 (20.6)
	**Fw**	18.4 (20.1)	1.04 (1.06)	24.8 (25.5)	70.9 (75.3)	
**Ref-40**	**Rv**	20.8 (21.9)	1.07 (1.11)	25.1 (26.0)	75.3 (76.9)	20.3 (21.5)
	**Fw**	20.2 (21.8)	1.06 (1.09)	24.9 (25.9)	76.0 (77.7)	
**2TI-0**	**Rv**	21.1 (22.3)	1.08 (1.11)	25.5 (26.0)	76.6 (79.2)	20.8 (21.7)
	**Fw**	20.6 (21.4)	1.07 (1.08)	25.4 (25.9)	75.9 (77.7)	
**2TI-40**	**Rv**	20.9 (21.7)	1.09 (1.10)	25.3 (25.7)	75.7 (78.5)	20.3 (21.0)
	**Fw**	20.0 (21.5)	1.07 (1.09)	25.1 (25.6)	75.5 (77.8)	
**4TmI-0**	**Rv**	20.5 (21.5)	1.07 (1.10)	25.2 (25.9)	75.7 (77.4)	20.2 (21.1)
	**Fw**	19.1 (20.5)	1.04 (1.07)	25.1 (25.7)	72.1 (75.6)	
**4TmI-40**	**Rv**	20.4 (21.7)	1.07 (1.10)	25.2 (25.8)	76.3 (79.0)	20.4 (21.5)
	**Fw**	18.3 (21.3)	1.04 (1.08)	25.0 (25.7)	70.9 (77.7)	

Table S2. Elemental ratios calculated from XPS peak profile fitting for Pb4f and N1s scans.

Sample	I/Pb	N (FA)/Pb	N (2TI_4TmI)/Pb
**Ref-0**	2.012	0.903	0
**2TI-0**	4.280	0.833	0.329
**2TI-10**	4.085	0.859	0.135
**2TI-40**	4.079	0.883	0.123
**4TmI-0**	3.965	0.946	0.265
**4TmI-10**	4.071	0.913	0.328
**4TmI-40**	4.264	0.618	0.688

Table S3. PbI_2 peak areas and Full Width Half Maxima (FWHM) calculated from GIWAXS intensity profiles.

Sample	Peak area	FWHM
**Ref-0**	7.00	0.038
**2TI-0**	1.79	0.033
**2TI-10**	5.30	0.038
**2TI-40**	1.62	0.038
**4TmI-0**	3.08	0.035
**4TmI-10**	5.96	0.037
**4TmI-40**	3.37	0.041

References

1. Wieghold, S. *et al.* Halide Heterogeneity Affects Local Charge Carrier Dynamics in Mixed-Ion Lead Perovskite Thin Films. *Chemistry of Materials* **31**, 3712–3721 (2019).

2. Sun, S. *et al.* Cage Molecules Stabilize Lead Halide Perovskite Thin Films. *Chemistry of Materials* **34**, 9384–9391 (2022).

3. Hidalgo, J. *et al.* Bulky cation hinders undesired secondary phases in FAPbI3 perovskite solar cells. *Materials Today* **68**, 13-21 (2023).

4. Gao, Y. *et al.* Molecular engineering of organic–inorganic hybrid perovskites quantum wells. *Nat Chem* **11**, 1151–1157 (2019).

5. Shi, E. *et al.* Two-dimensional halide perovskite lateral epitaxial heterostructures. *Nature* **580**, 614–620 (2020).
